# Prevalence and Associated Risk Factors of Urinary Tract Infection among Diabetic Patients: A Cross-Sectional Study

**DOI:** 10.3390/healthcare11060861

**Published:** 2023-03-15

**Authors:** Anas Elyas Ahmed, Suhaila Abdelkarim, Maria Zenida, Maisa Ali Hussein Baiti, Atyaf Abbas Yahya Alhazmi, Bushra Ahmed Hussain Alfaifi, Rania Qarmoush Mohammed Majrabi, Nidaa Qasem M. Khormi, Alyaj Alla Ali Hakami, Rafa Abdu Mohammed Alqaari, Raffan Ahmed Alhasani, Ramzi Abdu Alajam, Mohammed M. Alshehri, Aqeel M. Alenazi, Bader Alqahtani, Meshal Alshamrani, Ahmed Alhowimel, Siddig Ibrahim Abdelwahab

**Affiliations:** 1 Faculty of Medicine, Jazan University, Jazan GGGD6622, Saudi Arabia; 2 Department of Physical Therapy, College of Applied Medical Sciences, Jazan University, Jazan GGGD6622, Saudi Arabia; 3 Medical Research Centre, Jazan University, Jazan GGGD6622, Saudi Arabia; 4 Department of Pharmaceutics, College of Pharmacy, Jazan University, Jazan GGGD6622, Saudi Arabia

**Keywords:** diabetes mellitus, urinary tract infections, Saudi Arabia, risk factors

## Abstract

Urinary tract infections (UTIs) are one of the most common long-term complications of diabetes mellitus (DM). Additionally, various factors, such as socio-demographics, type of DM, fasting blood glucose, regular diabetes monitoring, comorbid chronic diseases, HbA1c, body mass index (BMI), and duration of DM, are also thought to predispose individuals to developing UTIs more frequently when they have DM. This research aims to evaluate the risk factors for UTIs and their prevalence among people with DM in Saudi Arabia (KSA). This cross-sectional study was conducted among 440 adults with type 1, type 2, and gestational DM. The participants had to be at least 18 years old, of both genders, and had been suffering from DM for any period of time. A self-administered questionnaire was utilized to collect data on demographic characteristics, such as sex, age, height, weight, material state, education level, income, and clinical profiles of DM and UTI. The crude (COR) and adjusted odds ratios (AOR) were calculated using logistic regression in the IBM SPSS software. The incidence of types 1 and 2 DM and gestational diabetes reached 34.1, 60.9, and 5%, respectively. Most of the participants had first-degree relatives with DM (65.9%). UTI was common in 39.3% of participants. A chi-squared statistical analysis revealed that the frequency of UTI varied depending (χ2 = 5.176, *P* = 0.023) on the type of DM. Burning urination and abdominal pain were the most common symptoms. The CORs for sex, marital status, hypertension, and BMI were significant (*P* < 0.05) and had values of 2.68 (95% CI = 1.78–4.02), 0.57 (95% CI = 0.36–0.92), 1.97 (95% CI = 1.14–3.43), and 2.83 (95% CI = 1.19–2.99), respectively. According to the adjusted model, only sex influenced the occurrence of UTIs. The AOR for sex was 3.45 (95% CI = 2.08–5.69). Based on this study, the authorities related to the health of DM patients can use its findings to guide awareness programs and clinical preparedness.

## 1. Introduction

Diabetes mellitus (DM) has recently been considered a growing health problem worldwide. In 2019, the global prevalence of DM was estimated to be 9.3% (463 million people); it is expected to rise to 10.2% (578 million) by 2030 and 10.9% (700 million) by 2045 [1,2]. In Saudi Arabia (KSA), the prevalence of DM has increased dramatically during the last few decades. At present, it is considered the most prevalent disease in the KSA [3]. The Saudi Health Interview Survey (SHIS) in 2013 showed that the total prevalence of diabetes was 14.8% and 11.7% for males and females, respectively. The prevalence increased with age and ranged from 7.8% among those aged 25 to 34 to 50.4% among those 65 and older. In men, one million were diabetic, 583,000 were on medication for diabetes, and 230,000 had uncontrolled diabetes. For women, 720,000 were diabetic, 367,000 were on medication, and 167,000 had uncontrolled diabetes [4,5].

Diabetic patients are at higher risk for all infections than non-diabetic patients, such as lower respiratory infections, UTIs, sepsis, endocarditis, skin infections, bone infections, joint infections, and mucous membrane infections [6]. Existing data indicate that the most common bacterial infection in diabetic patients is a UTI [7]. According to a study conducted in the KSA, the overall prevalence of UTIs in diabetic patients was 25.3%, 7.2%, and 41.1% in males and females, respectively. Many risk factors increase UTI frequency among diabetic patients [8,9]. Different studies confirmed that high blood glucose levels that are not adequately controlled could provide a rich source of nutrients for bacteria. Additionally, weakened immune systems in diabetic patients, such as decreased T-cell-mediated immune response and impaired bladder emptying due to autonomic neuropathy, may raise the risk of UTIs in diabetic patients since urine stays in the bladder for too long and becomes a breeding ground for bacterial growth [9,10]. Furthermore, among people with diabetes, females are at higher risk of UTIs than males due to their anatomy and reproductive physiology. A BMI greater than 30 kg/m^2^ was also discovered to be one of the risk factors associated with UTIs in diabetic patients in the KSA [10,11,12].

The fact that people with diabetes are more likely to suffer from UTIs and that the number of people with diabetes around the world has been growing in recent years may place a big financial strain on healthcare [13]. Further encouraging the development of an antibiotic-resistant urinary pathogen may be the high rates of antibiotic prescriptions, especially broad-spectrum antibiotics, for UTIs in these individuals [14,15]. UTIs in diabetic patients are more common and can lead to severe complications and potentially life-threatening conditions, such as renal papillary necrosis, renal or perirenal abscess, emphysematous pyelitis/cystitis, and emphysematous pyelonephritis, as well as urosepsis and bacteremia [12,16,17]. No studies have assessed the risk factors for UTIs among people with DM in the Jazan population in southwest of the KSA. Therefore, this study aimed to determine the prevalence of UTIs and the factors that put people with diabetes at an increased risk for developing UTIs in Jazan, as well as the incidence of UTI complications. In addition, a multivariate analysis was carried out to determine the causes of UTIs.

## 2. Materials and Methods

### 2.1. Study Design, Area, and Duration

For the current study, a cross-sectional design was appropriate for an observational epidemiological study. The investigation occurred at the Diabetes and Endocrinology Center in Jazan, KSA. In 2012, the center was launched to serve people with diabetes. The center offers numerous necessary services to its visitors. It includes a diabetes clinic for adults and children, an eye examination clinic for diabetes, a nerve and artery examination clinic, two health education clinics, two diabetic foot clinics, and a therapeutic nutritional clinic [18]. During the first six months of the 2020 calendar year, the center received 24,619 visits [18]. This study was conducted from December 2021 to June 2022.

### 2.2. Inclusion and Exclusion Criteria

The inclusion criteria were diabetic patients aged 18 years and older attending the Diabetes and Endocrinology Center in Jazan, KSA. Pregnant women, people under the age of 18, those with known urinary tract abnormalities, those who had recently used antibiotic therapy, those who had recently been hospitalized, and those who had surgery within the last four months were all excluded.

### 2.3. The Sample Size and Sampling Technique

The estimation was based on a sample size calculation for cross-sectional study design (n = (Z)2(1 − α)P(1 − P)/d2), using the following parameters: anticipated population proportion (P) = 50%, a 95% confidence level, and an error not greater than 5%. The sample size for this study was calculated to be 400 participants among the diabetic patients at the Diabetes and Endocrinology Center in Jazan. Additionally, we assumed a refusal rate of 10%. The data were collected from 440 participants. Simple random sampling was applied.

### 2.4. Data Collection and Study Measures

The data for this study were collected using a self-administered questionnaire. The questionnaire was designed in Arabic to be suitable for the participants. The questionnaire collected data about demographic characteristics, such as sex, age, height, weight, material state, education level, and income. Additionally, data about DM, such as type of DM, duration, glycemic profile, medications, adherence, and comorbidities, were acquired. The patients were also asked how often they had a UTI infection in the previous 12 months, as well as some risk or protective factors associated with UTIs, such as fasting glucose levels and HA1C.

### 2.5. Pilot Study and Pretesting

A pilot study was conducted with 20 participants to test if the questionnaire’s wording was clear and understandable. Each participant in this pilot study was asked to read and sign a consent form before data collection. The data from the pilot study were analyzed but not included in the main study.

### 2.6. Data Analysis

IBM SPSS version 23 was used for data entry and analysis. Means, standard deviations, frequencies, and percentages characterized the study variables. At a deeper level of data analysis, the chi-squared test was utilized to look for patterns. It was decided that a result was significant if the *P*-value was less than 0.05. Logistic regression modeling (LRM) was used to analyze the association between the dependent variable (UTI incidence) and the risk factors. The crude and adjusted odds ratios (OR) were obtained for all the independent variables. All variables included in the model were categorical except for age, which was incorporated as a continuous variable. The ORs were obtained with a *P*-value and 95% confidence intervals. The goodness of fit of the data to the multivariate logistic regression was tested using the Hosmer–Lemeshow test.

### 2.7. Ethical Considerations

Ethical approval was obtained from the Standing Committee for Scientific Research (REC-43/09/207). Additionally, informed consent was included in the questionnaire, and the participants’ consent was confirmed before data collection. Each participant was asked to read and sign a consent form before the start of data collection. The data did not include relevant participant details, such as name, file number, and telephone number.

## 3. Results

**Figure 1:**
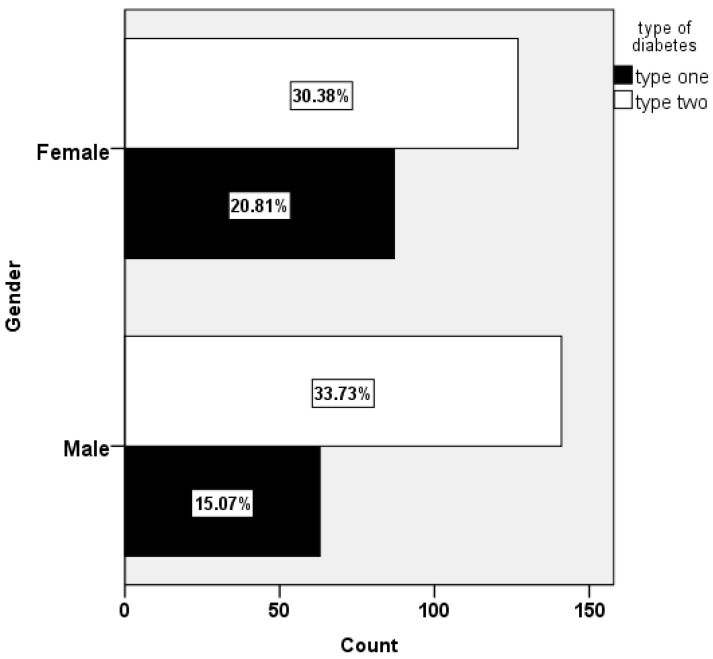
Distribution of types of diabetes by gender. Note that gestational diabetes is excluded from this chi-squared analysis.

A total of 440 participants were recruited from the Diabetes and Endocrinology Center in the Jazan region, KSA, of whom 46.6% were males, 53.3% were females, and 63.8% were married. More than two-thirds (69.5%) of the sample were over 35 years old, with a mean age of 44.36 and a standard deviation of 14.81. The percentage of university degree holders was 55.9%. Participants in the income range of SAR 5 to 15 K (USD 1 = SAR 3.766) made up more than half of the sample (55.4%). Participants with type 1 and type 2 DM and gestational diabetes were 34.1%, 60.9%, and 5%, respectively. One-third of the participants’ fathers held a high school diploma, while two-thirds of their mothers were illiterate. About 63.4% of the participants had a BMI over 26. More details are presented in Table 1. The distribution of diabetes types by gender is shown in Figure 1. A chi-squared statistical analysis reveals that disease rates vary depending (χ2 = 4.335, *P* = 0.037) on gender.

Most of the participants had first-degree relatives with diabetes (65.9%). Only 16.4% of the participants had no family history of diabetes among their first-degree relatives. The majority of participants (69.3%) are committed to a diabetes treatment, and about half (49.3%) regulated their blood sugar levels using pills. More details are depicted in Table 2.

**Figure 2:**
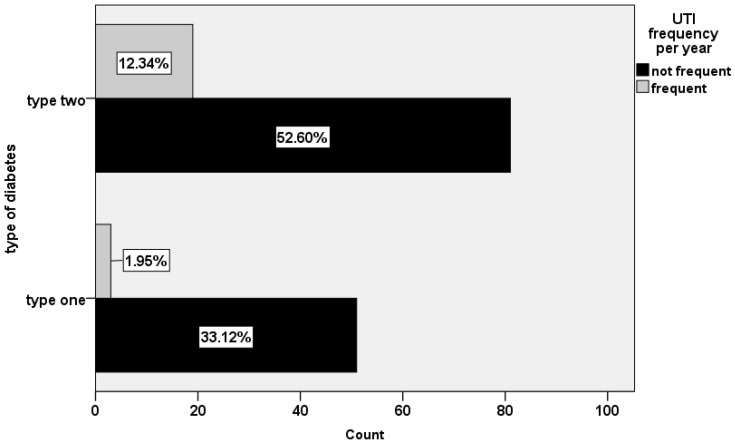
The frequency of UTIs among different types of DM (DM1 and DM2).

The prevalence of previous UTIs was 39.3%. Over half of the participants (60.6%) suffered from a UTI 1–2 times a year (Table 3). Approximately one-third of the participants experienced complications from a urinary tract infection. About 37% of the participants experienced UTI complications. Burning urination and abdominal pain were the most common symptoms. The distribution of the frequency of UTIs by type of DM is shown in Figure 2. A chi-squared statistical analysis reveals that the frequency of UTIs varies depending (χ2 = 5.176, *P* = 0.023) on the type of DM (Table 3).

A logistic regression model was conducted to obtain the crude odds ratio (COR) and adjusted odds ratio (AOR) for all the included factors (gender, marital status, educational level, income, type of DM, fasting blood glucose, regular DM checking, chronic diseases, HbA1c, BMI, and duration of DM). The dependent variable is the diabetic patients’ previous exposure to a UTI. A univariate linear regression model was conducted to calculate the COR and its statistical significance for each independent variable separately. Without adjusting or adding all the variables in the multivariate logistic model, the initial results show that sex, marital status, chronic diseases, and BMI are individually and statistically significant factors (Table 4). The CORs for sex, marital status, hypertension, and BMI are 2.68 (95% CI = 1.78–4.02), 0.57 (95% CI = 0.36–0.92), 1.97 (95% CI = 1.14–3.43), and 2.83 (95% CI = 1.19–2.99), respectively. Then, a multivariate logistic regression model was used to calculate the AORs. However, before that, it was necessary to ensure the validity of the data to be included in the model using the Hosmer–Lemeshow test. The goodness of fit of our data to the multivariate logistic regression was assured (*P* > 0.05). Including all independent variables in the multivariate logistic regression model led to the adjustment of some values of the ORs. It is found that sex is the only factor that shows statistical significance (Table 4). The AOR for sex is 3.45 (95% CI = 2.08–5.69).

## 4. Discussion

The purpose of this study, which is the first of its kind, was to evaluate the prevalence of urinary tract infections (UTIs) among diabetic patients in Jazan, KSA, using a cross-sectional study and to analyze the risk factors associated with UTIs using logistic regression modeling. In the KSA, the prevalence of DM has increased dramatically during the last few decades. At present, it is considered the most prevalent disease in the KSA [3]. According to a study conducted in the KSA, the overall prevalence of UTIs in diabetic patients was 25.3%, 7.2%, and 41.1% in males and females, respectively [4,5]. The current study employed a representative sample of diabetic patients (Table 1).

Together, the growing number of diabetic patients with UTIs and the growing number of people with diabetes around the world in recent years may place a big financial strain on healthcare [13]. According to the Saudi Health Interview Survey (SHIS), which was conducted in 2013, 14.8% of men and 11.7% of women had diabetes. This difference was seen between the sexes [3,8]. Figure 1 shows that the current findings align with the previous report [3,8]. The chi-squared statistical analysis reveals that disease rates vary depending (χ2 = 4.335, *P* = 0.037) on the gender of the diabetic patients. It should be noted that this chi-squared analysis did not include gestational diabetes.

According to the World Health Organization and the American Diabetes Association’s respective standards, the prevalence of gestational DM has grown in many racial and ethnic groups over the previous 20 years [19]. As reported in this study, the prevalence of gestational DM is 5% in the Jazan region, KSA. The overall prevalence of gestational DM is 13.2% in Germany [20], 18.9% and 17.8% in India [21], 8% in Egypt [22], 12.5% in Riyadh, KSA [23], 18.7% in Abha, KSA [24], 6.4% in Qatar [25], 24.5% in Morocco [26], and 3.7% in China [27]. In Australia, it was discovered that women whose country of origin was China or India had a greater frequency of gestational DM than women whose county of birth was Europe or Northern Africa [28].

The increased risk of UTIs among diabetic patients and the rise in DM prevalence worldwide in recent years may place a significant financial burden on healthcare [13]. Existing data indicate that the most common bacterial infection in diabetic patients is a UTI [7]. The prevalence of UTIs is 39.3%. More than half (60.6%) of the participants in the current research suffer from a UTI 1–2 times each year. A study conducted in the KSA found that the overall prevalence of UTIs in diabetic patients was 25.3% [8]. The prevalence of diabetic UTIs was reported to be 13.8% in Ethiopia [29], 17.5% in India [30], and 9.71% in the USA [31].

A logistic regression model was used to obtain the COR and AOR for each component. Each independent variable’s COR and statistical significance were calculated using a univariate linear regression. The initial results show that sex, marital status, chronic illnesses, and BMI are statistically significant contributors (Table 4). The CORs for sex, marital status, hypertension, and BMI are 2.68, 0.57, 1.97, and 2.83, respectively. According to a study from the KSA, female sex, hypertension, insulin treatment, a body mass index (BMI) of more than 30 kg/m^2^, and nephropathy all raise the incidence of UTIs in diabetic patients [8]. The AORs were calculated using a multivariate logistic regression, and all independent variables were included in the multivariate logistic regression to obtain the adjusted OR results. Sex is the only statistically significant factor (Table 4). The AOR is 3.45 (95% CI: 2.08–5.69) for sex. According to previous research using administrative data from the US population, women had a significantly higher annual incidence of UTIs than men (12.9% vs. 3.9%) [32]. In addition, studies with a similar geographic population to ours have indicated the predisposition of the female gender to UTIs [33].

Many variables have been linked to UTIs in people with diabetes in previous studies. Their findings indicate that sex, educational attainment, and a UTI history are predictors. These outcomes are consistent with research from Saudi Arabia [8], China [34], Kuwait [35], and the United States [32]. Due to anatomical differences in the urinary system, women typically have more UTIs than men, which may account for the disparity between the sexes. The lower levels of typical vaginal flora (Lactobacilli), the less acidic pH of the vaginal surface, the poor sanitary conditions, the short and wide urethra, and the proximity to the anus may all contribute to the increased infection rates of UTIs in our study’s female participants.

Most patients had HbA1c values of seven or more; these levels were unrelated to the patients’ UTI status, either positive or negative, and had only a weak correlation. This supports the finding from a meta-analysis of 22 studies that the degree of HbA1c derangement does not necessarily impact the biological flora or play any role in UTI susceptibility [36].

Due to the limitations of the cross-sectional study design, it was not possible to find out what caused certain factors to be linked to UTIs in the sample population. Additionally, this study did not look at factors such as where people lived, if they smoked, or how much alcohol they drank, which could have changed the results. One of the drawbacks is that this study was restricted to one province. It might only represent some Saudis, making the generalization of the study’s findings challenging.

## 5. Conclusions

The analysis reveals that UTI rates vary depending on the gender of the diabetic patients and that the frequency of UTIs varies depending on the type of DM. In conclusion, the present study shows that the female gender has a higher risk of UTIs among people with diabetes. Based on this study, the authorities related to the health of diabetic patients can use its results to guide awareness programs and clinical preparedness. The findings can be helpful for high-risk patients in selecting the most appropriate infection prevention strategies.

## Figures and Tables

**Table 1:** Background characteristics of the participants.

Variables	N (%)			
	Categories	Male	Female	Total
Age groups	Less than 35	50 (11.4)	84 (19.1)	134 (30.5)
	36–55	104 (23.6)	100 (22.7)	204 (46.3)
	More than 56	51 (11.6)	51 (11.6)	102 (23.2)
Marital status	Married	151 (34.3)	130 (29.5)	281 (63.8)
	Single	43 (9.8)	69 (15.7)	112 (25.5)
	Divorced	8 (1.8)	10 (2.3)	18 (4.1)
	Widow	3 (0.7)	26 (5.9)	29 (6.6)
Educational level	Illiterate	8 (1.8)	36 (8.2)	44 (10.0)
	Primary	6 (1.4)	20 (4.5)	26 (6.9)
	Intermediate	12 (2.7)	16 (3.6)	28 (6.3)
	Secondary	47 (10.7)	49 (11.1)	96 (21.8)
	University	132 (30.0)	114 (25.9)	246 (55.9)
Educational level of father	Illiterate	85 (19.3)	91 (20.7)	176 (40)
	Secondary	70 (15.9)	75 (17.0)	145 (33.0)
	University	42 (9.5)	60 (16.6)	102 (23.2)
	Higher studies	8 (1.8)	9 (2.0)	17 (3.9)
Educational level of mother	Illiterate	134 (30.5)	140 (31.8)	247 (62.3)
	Secondary	45 (10.2)	54 (12.3)	99 (22.5)
	University	23 (5.2)	37 (8.4)	60 (13.6)
	Higher studies	3 (0.7)	4 (0.9)	7 (1.6)
Family income	Less than 5 k	32 (7.3)	71 (16.1)	103 (23.1)
	5 to 10 k	53 (12.0)	70 (15.9)	123 (27.9)
	10 to 15 k	62 (14.1)	59 (13.4)	121 (27.5)
	More than 15 k	58 (13.2)	35 (8.0)	93 (21.2)
Type of diabetes	Type one	63 (14.3)	87 (19.8)	150 (34.1)
	Type two	141 (32.0)	127 (28.9)	268 (60.9)
	Gestational DM	1 (0.2)	21 (4.8)	22 (5.0)
BMI	Less than 25	69 (17.2)	78 (19.4)	147 (36.6)
	26–35	102 (25.4)	109 (27.1)	211 (52.5)
	More than 36	17 (4.2)	27 (6.7)	44 (10.9)
Duration of DM	Less than 5 yrs.	75 (17.0)	105 (23.9)	180 (40.9)
	6–10 yrs.	57 (13.0)	53 (12.0)	110 (25.0)
	11–20 yrs.	60 (13.6)	54 (12.3)	114 (25.9)
	More than 21 yrs.	13 (3.0)	23 (5.2)	36 (8.2)
Total		205 (46.6)	235 (53.6)	440 (100)

**Table 2:** Characteristics of DM among the participants.

Variables	Categories	N	(%)
Family history of DM	First degree	290	65.9
	Second degree	78	17.7
	None	72	16.4
Adherence to diabetes management	Yes	305	69.3
	Sort of	116	26.4
	Never	19	4.3
Type of diabetes management	Insulin	94	21.4
	Pills	217	49.3
	Pills and insulin	72	16.4
	Insulin pump	4	0.9
	Injections	15	3.4
	Diet and Exercise	38	8.6
Adherence to diabetes monitoring	Yes	219	49.8
	Sort of	190	43.2
	Never	31	7.0
Fasting glucose	80–130	196	44.5
	130–200	179	40.7
	More than 200	65	14.8
Normal HB A1C	Yes	293	66.6
	No	147	33.4
Follow-up	Less than 2	122	27.7
	3–5	193	43.9
	More than 5	89	20.2
	No visits	36	8.2
Chronic diseases	None	230	52.4
	Hypertension or CVD	73	16.6
	Lipids or obesity	45	10.3
	Lipids and HTN or CVD	91	20.7

**Table 3:** Characteristics of UTI among the participants.

Variables	Categories	N	(%)
Previous UTI	Yes	173	39.3
	No	267	60.7
No. of UTIs per year	1–2	97	60.6
	3–4	41	25.6
	5 and more	22	13.8
Symptoms of UTI (Yes)	Burning micturition	117	26.6
	Frequent micturition	84	19.1
	Abdominal pain	97	22.0
	Loin pain	71	16.1
	Fever	27	6.1
	High fever	32	7.3
	Nausea and vomiting	17	3.9
Complications of UTI	Yes	64	37.0
	No	109	63.0

**Table 4:** Multivariate logistic regression.

Factors	Categories	COR	95% CI for COR		AOR	95% CI for AOR	
			Lower	Upper		Lower	Upper
Gender	Male (ref)						
	Female	2.68 *	1.78	4.02	3.45 *	2.08	5.69
Marital status	Married (ref)						
	Single	0.57 *	0.36	0.92	0.60	0.32	1.13
	Divorced	0.73	0.26	2.04	0.55	0.17	1.76
	Widow	1.44	0.67	3.09	0.78	0.28	2.20
Educational level	Illiterate (ref)						
	Primary	1.051	0.39	2.85	0.74	0.22	2.48
	Intermediate	0.535	0.20	1.41	0.39	0.11	1.34
	Secondary	0.427	0.20	0.90	0.74	0.27	2.01
	University	0.496	0.26	0.96	0.72	0.28	1.90
Income	Less than 5 k (ref)						
	5 to 10 k	1.02	0.59	1.76	1.15	0.57	2.30
	10 to 15 k	0.99	0.57	1.71	1.75	0.83	3.69
	More than 15 k	0.74	0.41	1.33	1.24	0.55	2.79
Type of DM	Type I (ref)						
	Type II	1.22	0.81	1.84	1.01	0.59	1.71
Fasting blood glucose	80–130 (ref)						
	130–200	1.65	0.92	2.94	0.97	0.56	1.69
	More than 200	1.09	0.71	1.68	1.41	0.66	2.98
Regular DM checking	Yes (ref)						
	Sort of	1.314	0.87	1.98	1.34	0.82	2.20
	Never	0.94	0.43	2.06	0.85	0.32	2.28
Chronic diseases	NA (ref)						
	Hypertension or CVD	1.97 *	1.14	3.43	1.80	0.91	3.53
	Lipids or obesity	2.42 *	1.25	4.68	1.94	0.89	4.21
	Lipids and HTN or CVD	2.07 *	1.25	3.43	1.71	0.89	3.27
HbA1c	Controlled (ref)						
	Uncontrolled HbA1c	1.40	0.90	2.20	1.26	0.73	2.19
BMI	Less than 25 (ref)						
	26–35	1.89 *	1.19	2.99	1.54	0.92	2.59
	More than 36	2.83 *	1.41	5.71	2.03	0.90	4.56
Duration of DM (yrs)		1.0	0.977	1.03	1.04	0.81	1.33

NA: Not available; COR: Crude odds ratio; OAR: Adjusted odds ratio; HbA1c: Hemoglobin A1c; Ref: Reference group; CI: Confidence intervals; BMI: Body mass index; K: 1000; *: Significant at 0.05. The dependent variable is diabetic patients’ previous exposure to a urinary tract infection.

## Data Availability

To preserve the confidentiality of the participants’ data and in the implementation of the commitment between the research team, the ethics committee, and the participants, the research team is unable to share the research data for this study.
